# Joint influence of genetic origin and climate on the growth of Masson pine (*Pinus massoniana* Lamb.) in China

**DOI:** 10.1038/s41598-020-61597-9

**Published:** 2020-03-13

**Authors:** Zhen Zhang, Guoqing Jin, Zhongping Feng, Linshan Sun, Zhichun Zhou, Yi Zheng, Chengzhi Yuan

**Affiliations:** 1 Research Institute of Subtropical Forestry, Chinese Academy of Forestry, Hangzhou 311400, Daqiao Rd 73, Fuyang area, Hangzhou, 311400 P. R. China; 2 Zhejiang Provincial Key Laboratory of Tree Breeding, Daqiao Rd 73, Fuyang area, Hangzhou, 311400 P. R. China; 3 Laoshan Forest Farm of Chun’an Country, Zhejiang Province, Chun’an, 311700 China; 4 Forest farm administration in Hubei taizi mountain, Jingshan, 431822 China

**Keywords:** Ecological genetics, Plant breeding

## Abstract

Adaptive of trees and its correlation with the climatic are causing changes in tree species performance and distribution, which will change breeding programs and influence forest productivity. To further evaluate the joint influence of climatic factors and provenance on the ring width (RW) and ring density (RD) of Masson pine. We selected 18 provenances at Chun’an (CA) and Taizi Mountain (TZS) test site, which representing four different breeding regions, including the south, west, north and east-central regions. The results showed that the provenance effects were significantly for the RW and RD. The provenances from high temperature and low latitude regions had greater mean RW compared to species from local and cold sources. The geographical genetic variation in wood traits is generally weak. The correlation between RW of Masson pine and precipitation was stronger in the relatively arid TZS site compared with that in relatively wet CA site, as well as the effect of temperature and precipitation on RD was earlier than that in CA test site. The response relationship between establishing the width of tree rings and the environmental variables of provenance indicated that during the transition from the northern and western breeding regions to the eastern and southern breeding regions, the response of RW to climate factors changed from being temperature-based to being precipitation-based. In addition, the response of provenance to the climate of seed sources origin showed their own variation characteristics in each breeding area. Therefore, genetic improvement of big diameter wood and wood density can be gain through selection of provenance and analysis of adaptability.

## Introduction

Diameter growth and wood density are important selection traits for sawn timber and pulpwood, respectively, which have been part of the long-term breeding program of conifer species^1,2^. The research showed that trees could adapt to the rapid environmental changes through genetic selection and showed different responses to environmental changes in radial growth and wood density^3–5^. In addition, there is strong growth correlation of trees with climate, particularly with temperature and precipitation^6,7^. The different tissues cells of wood change at different growing season, affect the ring width (RW hereafter) and ring density (RD hereafter) parameters and involve fast and slow of growth processes^8^. These xylogenesis phases (cell expansion, tracheid maturation and cell-wall thickening and lignification) translate into varied responses of radial growth and wood property to climate^9,10^. The coupling relationship will be found among density parameters or between RW and climate, although such changes often depend on how the RW and wood density change over time as a function of genetics, site condition and climate factors^9,11–13^.

The distribution area of Masson pine (*Pinus massoniana* Lamb.) in China is located at latitude 21 °41′∼33 °56′N and longitude 102 °10′∼123 °14′E. The distance between the north and south provenances is approximately 1360 km, and the distance between the east and west provenances is approximately 1900 km^14^. The genetic improvement work of Masson pine have been implemented since the 1970s in China, which is focus on survival, rapid growth, resin production, stem form and wood quality. Among the vast natural distribution regions of Masson pine, the southern provenances of Masson pine grows fast and the provenances in Guangdong and Guangxi have large radial growth and “polycyclic” annual shoots^15^. Compared with the southern provenances, the northern provenances, which have a relatively short growing season and fewer precipitation, temperature and light resources, especially in regions where soil moisture is relatively low, limits the radial growth and the formation of wood characteristics in Masson pine.

In the future, the main limiting factors of tree growth were the length of drought periods and temperature variations^16–19^,which affecting the adaptability of populations and the distribution of species. Studies have shown that changes in temperature and precipitation obviously affect the RW hereafter and RD of Masson pine. For example, the mean temperature from January to May significantly affected the growth in the Yangming Mountain area, Hunan Province, China, and precipitation during the previous growing season had a lagged effect on the RW of Masson pine was reported in 2017 by Jiang *et al*.^20^.

Similar to other conifer species, radial growth of the stem and wood quality of Masson pine, are also influenced by cambial age as well as by genetic origin. Wood density showed to be controlled by heritability above medium level, which provided the possibility of screening superior provenances or lineages^21–23^. For example, the growth rate of major conifer species could be improved by provenance selection, operation and management, which would not affect the wood density^24–28^. Meanwhile, the adaptability of future forests depends on the response of genotypes to rapidly changing climatic conditions^29^. Genotype determines the genetic quality, and variations caused by genotype can be inherited; thus, growth traits and wood quality can be improved by genetic improvements^30^.

Provenance tests and provenance selection are the most basic tools for the genetic improvement of forest trees. Adaptive genetic variations of provenance and their correlations with climatic factors have been investigated in experiments, and reports have suggested that genetic variations should be considered for the provenance selection as an adaptation to climate change^31–36^. Given the above background, this study described the range of intraspecific variation of RW and RD and analyzed the effects of different sites, provenances and climatic factors on the RW and RD growth. The objectives were: (1) analyze the RW and RD among provenances at different testing sites; (2) test the effects of provenances and climate on RW and RD; and (3) assess the influence of climate change on the adaptive of Masson pine in China.

## Materials and Methods

### Site and plant material

**Figure 1:**
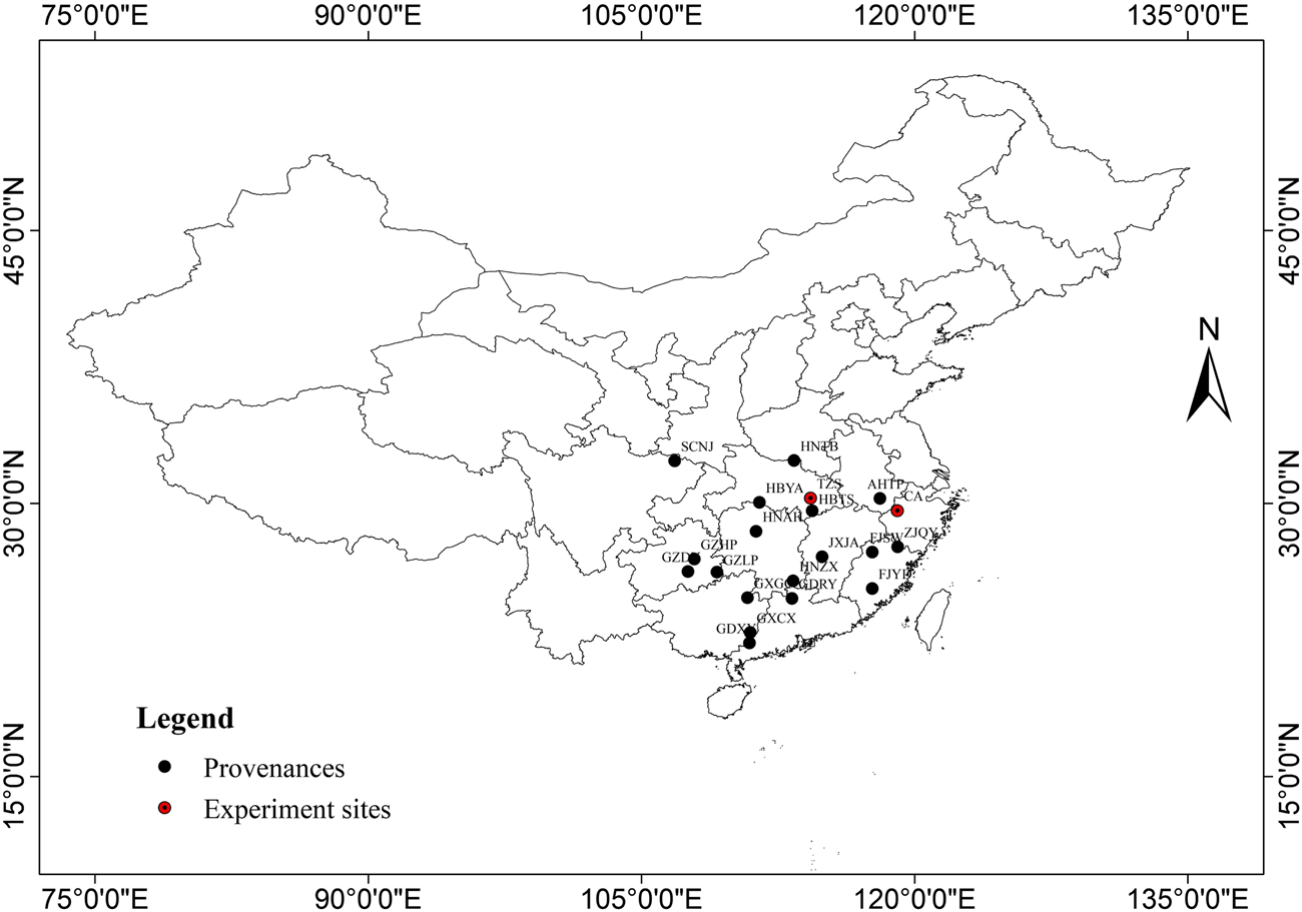
Geographic locations of origin of the studied Masson pine provenances.

The test materials for this study came from two provenances test forests established in 1984. One forest was located in Chun’an town (N29°33′, E119°03′; referred to as CA hereafter) of Zhejiang Province, and a total of 49 provenances from 14 provinces were tested in this forest. The experiment used completely randomized block design (8 blocks and 8-tree plots planted with a 2-row layout and 4 trees per row in each blocks). The row spacing of the initial planting was 2 m × 2 m, and thinning was done in the 10^th^ and 15^th^ year after afforestation. The other trial was in a forest at Taizi Mountain (N30°20′, E114°18′; referred to as TZS hereafter), Hubei Province (Fig. 1), and the same number of provinces as the CA test sites was implemented, a total of 69 provenances. The experiment used completely randomized block design (9 blocks and 4-tree plots planted with a 1-row layout and 4 trees per row in each blocks). The row spacing of the initial planting was 2 m × 2 m, and thinning was done in the 18^th^ year after afforestation.

The condition of CA and TZS site is different. TZS site is in a relatively arid area with annual precipitation of 1090 mm and an mean annual temperature of 16.4 °C, whereas CA presents annual precipitation of 1530 mm and an mean annual temperature of 17.2 °C. The warmest month in the two study regions is July, and the coldest month is January. The total precipitation in CA occurs mainly from February to August, which accounts for 71% of the annual precipitation. The total precipitation in TZS occurs mainly from April to August, which accounts for 65% of the annual precipitation (Fig. S1). The same 18 provenances were selected from each site, and the provenances were distributed in the northern, central-eastern, southern and western regions of the natural distribution range of Masson pine, which represented different distributions of hydrothermal resources (Table 1). (The northern regions of the Masson pine natural distribution area are mainly in the Dabie Mountains and Daba Mountains, the southern regions are mainly distributed south of the Wuyi Mountains and Yunkai Mountains, the central-eastern regions are mainly distributed in the Wuyi Mountains and north of the Nanling Mountains, and the western regions are mainly distributed south of the Qinling and Hengduan Mountains). The geographic coordinates and climatic factors of the 18 provenances are shown in Table 1.

**Table 1:** Statistical description of geographic coordinates, temperatures, precipitation and expressed population signal value for 18 provenances at CA and TZS test sites.

Provenances	Latitude (°)	Longitude (°)	Altitude (m)	Region	MAT (°C)	P (mm)	RW value (mm)		RD value (g/cm^3^)		Dt_1.3_ value (cm)		EPS (RW)		EPS (RD)	
							CA site	TZS site	CA site	TZS site	CA site	TZS site	CA	TZS	CA	TZS
GDXY	22.35	110.93	141	South	22.9	1785	4.21	4.00	0.28	0.20	12.79	12.83	0.89	0.90	0.87	0.96
GXCX	22.92	110.97	99	South	21.9	1762	5.21	4.49	0.28	0.25	15.78	13.86	0.96	0.87	0.98	0.88
GDRY	24.78	113.27	98	South	20.4	1635	4.72	4.42	0.23	0.26	13.97	13.26	0.87	0.86	0.87	0.87
GXGC	24.83	110.82	169	South	17.8	1752	4.19	4.31	0.30	0.26	13.44	14.07	0.92	0.91	0.97	0.94
FJYD	25.33	117.67	226	South	20.4	1654	4.84	3.44	0.26	0.26	14.29	10.43	0.85	0.93	0.91	0.91
FJSW	27.33	117.67	218	South	18.3	1888	4.46	3.62	0.21	0.22	13.84	10.83	0.93	0.88	0.94	0.89
GZLP	26.23	109.15	568	West	16.7	1469	3.26	3.75	0.22	0.25	9.93	11.47	0.94	0.85	0.90	0.86
GZDY	26.27	107.55	969	West	16.5	1153	4.05	3.91	0.19	0.21	11.87	11.74	0.86	0.87	0.89	0.89
GZHP	26.95	107.90	835	West	16.2	1202	3.70	3.41	0.28	0.27	11.10	9.61	0.88	0.89	0.87	0.94
SCNJ	32.35	106.83	579	West	16.1	1086	3.35	3.77	0.25	0.29	10.15	10.38	0.85	0.90	0.87	0.94
HNZX	25.75	113.33	139	Central–eastern	18.6	1488	3.92	3.56	0.26	0.25	11.86	10.36	0.85	0.93	0.95	0.97
ZJQY	27.62	119.07	400	Central–eastern	16.2	1787	4.00	3.26	0.23	0.21	12.13	10.65	0.86	0.86	0.89	0.92
JXJA	27.08	114.92	71	Central–eastern	18.8	1549	3.76	3.24	0.20	0.27	11.19	7.32	0.90	0.89	0.94	0.91
HNAH	28.47	111.30	128	Central–eastern	16.5	1741	2.87	3.48	0.31	0.21	8.55	11.42	0.85	0.91	0.88	0.94
HBTS	29.60	114.38	78	Central–eastern	16.1	1525	3.67	3.20	0.21	0.23	11.07	10.29	0.86	0.93	0.87	0.92
HBYA	30.07	111.48	74	North	15.9	1157	2.98	3.48	0.24	0.27	9.29	10.81	0.87	0.85	0.91	0.90
AHTP	30.28	118.10	59	North	8.9	1866	3.75	3.59	0.22	0.26	11.27	10.86	0.90	0.87	0.92	0.87
HNTB	32.37	113.38	68	North	14.3	924	3.22	3.31	0.27	0.21	9.93	10.75	0.91	0.88	0.94	0.89

^†^MAT, P, RW, RD, Dt_1.3_ and EPS represent mean annual temperature, annual total precipitation, ring width, ring density, diameter cumulative growth and expressed population signal, respectively.

### Sampling and analysis

In November 2016, sampling of the experimental forests was carried out in three blocks at two test sites, respectively. From each provenance, a total of 6 trees in three blocks were selected in each site. 216 trees were selected from the two sites, and 5.15-mm cores were extracted at 1.3 m using a increment borer^3^. Resin composition in the core samples was extracted by Soxhlet extractor^37^. The cores were removed, dried, and polished step-by-step with different grades of sandpaper (400, 800, 1200 and 1500 mesh). The cell lumina and cell wall of the wood were clearly showed under 20x microscope, and the RW was measured using a LINTAB^Tm^6.0 RW measuring instrument (accuracy 0.01 mm) (LinTab 6.0, Rinntech, Heidelberg, Germany). The X-ray radiographic images were analyzed with density software to determine RD (g/cm^3^) profiles of each specimen^38^. The diameter cumulative growth (Dt_1.3_) was calculated according to the RW^3^.

### Data processing

We tested the homogeneity of variance of RW, RD and cumulative stem growth^39^, and used the following mixed linear model for variance analysis^38^:

1 $${{y}}_{{ijklm}}={u}+{{S}}_{{i}}+{B}{({S})}_{{j}({i})}+{A}_{{m}}+{P}{(A)}_{k({m})}+{S}\ast {P}{(A)}_{{ik}({m})}+{S}\ast {A}_{{ikm}}+{{\varepsilon }}_{{ijklm}}$$

where *y*_*ijklm*_ represented the value of the *m*th year the *l*th tree of the *k*th provenance growing at the *j*th block within the *i*th site; and A_m_, *S*_*i*_, *B*(*S*)_*j*(*i*)_, *P*(A)_*k*(*m*)_, S*P(A) _*ik*(*m*)_, S*A_*im*_ and *ε*_*ijklm*_ represented the effects of *m*th tree age, the *i*th site, *j*th block within *i*th site, *m*th year within *k*th provenance, interaction between the *i*th site and *m*th year within *k*th provenance, interaction between the *m*th tree age and *i*th site and the error, respectively. *B*(*S*)_*j*(*i*)_ and *ε*_*ijklm*_ were considered as random factor and the rest was considered as fixed factor. In addition, the affect of provenance-by-block interactions and tree age-by-block interactions was little, which could be ignored in the model^40^. The least square method was used to estimate the first order auto-correlation of model residuals^38,40^.

The statistical results of cross dating of RW were tested by the program of COFECHA^41^. The results showed that the main data series covered 30 years from 1987 to 2016 (corresponding to rings 4 to 33). RW and RD were not only affected by environmental and ecological factors but also controlled by their own physiological factors; therefore, the growth trends of trees would be removed before application. The RW and RD series were standardized using ARSTAN software with the negative exponential declination method. This method eliminated the influence of low-frequency variations of the RW and RD^42^. The RW and RD sequences that removes the growth trend will be used to create the residual chronologies^43^ (RES). The basic statistics of the remaining chronology of the rings are listed (Table S1), including the mean sensitivity, the mean sensitivity, the mean inter-serial correlation, frist-order autocorrelation, express population signal^44^.

The meteorological data period was 1986∼2016, and was downloaded from the China Meteorological Science data sharing service network (http://cdc.cma.gov.cn). Jingshan weather station (N31°03′, E113°58′, altitude 85 m) 70 km away from TZS test point and Chun ‘an weather station (N39°37′, E119°01′, altitude 171 m) 10 km away from CA test site were selected, respectively. For the climate stations of provenance, the same climate stations were chosen to provide seed sources in the report.

We analyzed the response function of growth rings and climate factors. The monthly mean temperature and total precipitation were transformed into 24 independent variable at the two test sites, respectively. The relationship between tree climate condition and radial growth was evaluated by regression and principal component analysis of chronology and climate variables^43^. Considering the growth of tree was affected by the lagging effects of climate change in the previous growing season^44^. Thus, the selection of meteorological data was from November of last year to the following October^29^.

The multivariate regression analysis was done to test the influence of climate on growth of Masson pine. Therefore, to obtain the optimal equation, the stepwise regression method was used to screen the independent variables included in the equation, and adjusted *R*^2^. The linear relationships of the predictions were also compared. In addition, the slope differences among the provenances were first tested. Subsequently, we compared the differences between the intercepts. The statistical significance of these tests among compared provenances was set at a probability value (*P*) of 0.05.

Five temperature, three precipitation and geographic coordinates variables were chosen. They included the following: mean temperatures (annual, January, from May to August), mean minimum and maximum daily temperature, annual total precipitation, precipitation in January, mean precipitation from May to August, and geographic coordinates (latitude and longitude). The differences between the above mentioned variables of origin and planting place of each provenance were used to analyze the trend of climate factors after each provenance introduced to the planting place. We calculated Principal Component Analysis (PCA) of width and density characteristics, respectively. The PCA axes which accounted for at least 50% of the total variance were kept in the PCAs^29^.

## Results

### Differences in provenance for RW, RD

**Figure 2:**
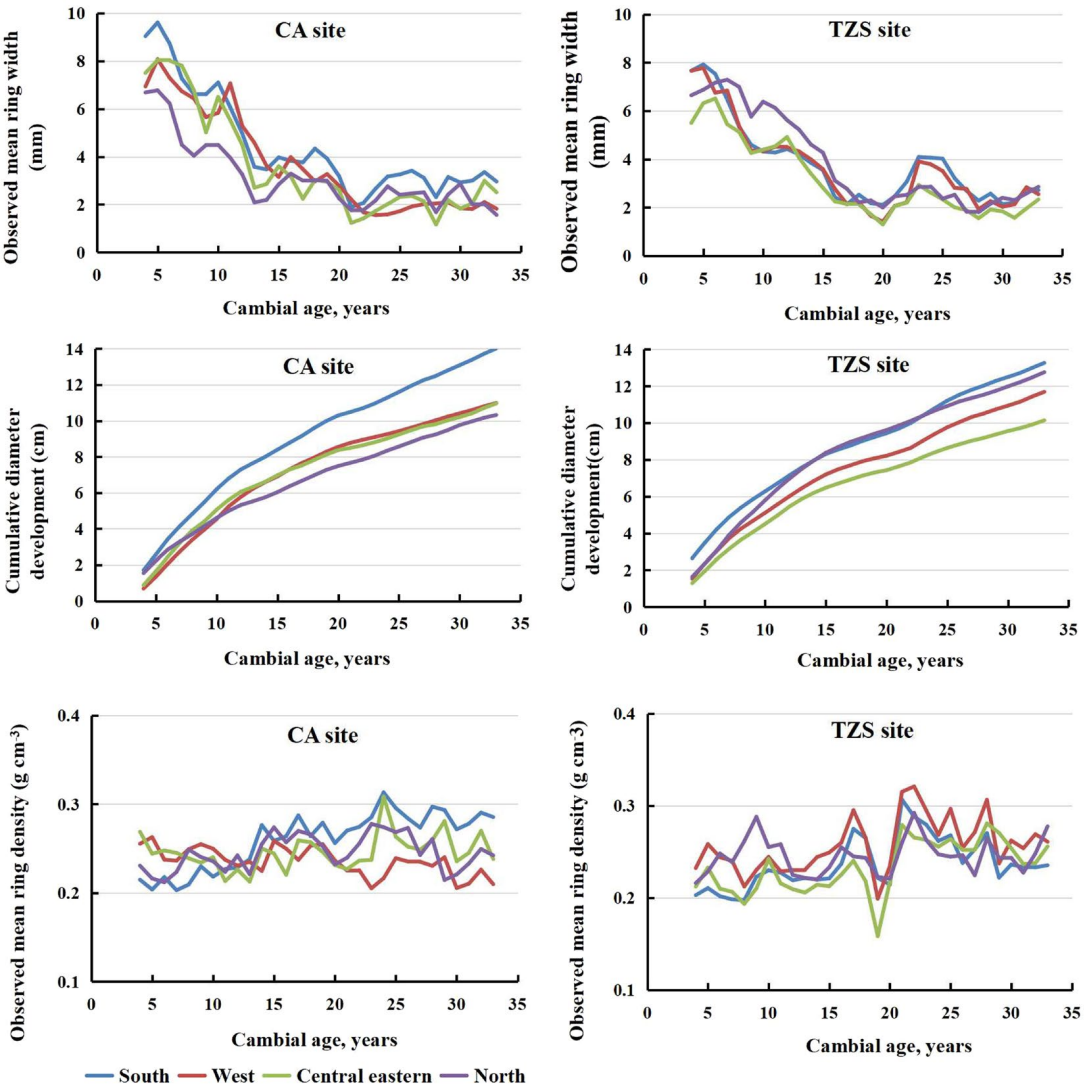
Observed mean RW, RD and D_1.3_ from pith to bark in four breeding regions at CA and TZS test site of Masson pine.

The differences in the mean RW between provenances were statistically significant, which showed that the RW increase was significantly affected by genetic factors. The mean RW of the provenances closest to the CA and TZS sites, AHTP and HBTS, were approximately 28.02% and 28.73% lower than that in the best performing provenance (GXCX), respectively (Table 1). Although the mean annual RW of the southern regions provenances was higher than that in the other production regions, the relative differences in the RW production interval varied with age, and these differences formed in the early stages of growth (Fig. 2). In addition, RW continued to increase first and then decreased at CA test site in the two periods of 10–15 years old and 15–20 years old, while TZS test site appeared in 20–25 years old. The change was related to the thinning of the experimental forest during that time period. The Dt_1.3_ differences between the provenances were statistically significant. The differences in Dt_1.3_ between the breeding regions changed with the age of the trees (Fig. 2). Similar to RW and Dt_1.3_, the differences in mean RD between species were statistically significant, and the mean RD showed a strong provenance effect (Table 1). The difference in mean RD for each production interval varied with age, and this difference fluctuated and occurred throughout the entire growth period (Fig. 2).

### Relationship between chronologies and climate

**Figure 3:**
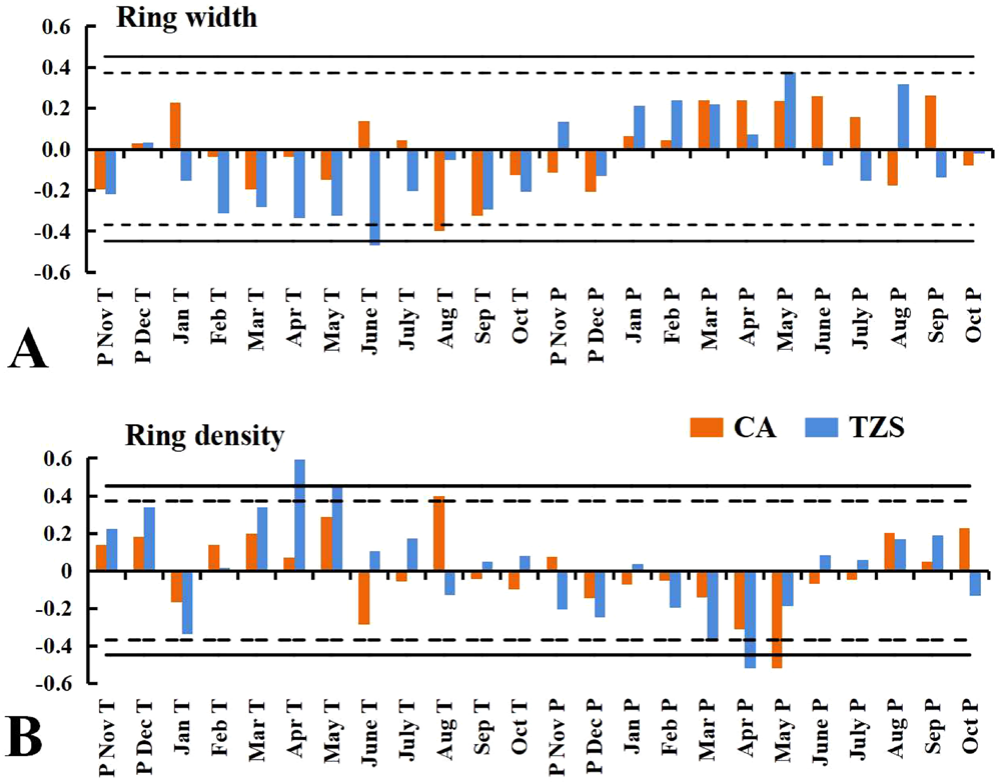
Response coefficient of the RW (**A**) and the RD (**B**) with monthly mean temperature (T) and precipitation (P) for the previous year (pNov–pDec) and the current growing season (Jan–Oct). ^†^ dotted line shows statistically significant correlation, p < 0.05 and solid line p < 0.01.

At CA test site, the RW of Masson pine showed significant negatively correlations to the average temperature in August. At TZS test site, the correlation between RW and mean temperature in June, was also negative, and showed significant positively correlations to precipitation in May (Fig. 3A).

At CA site, RD showed negatively correlations to precipitation in May and positively correlations to the average temperature in August. At TZS test site, RD was also significant negatively correlated with the precipitation in April and positively correlated with the mean temperature from April to May. At TZS test site, the time period in RD was affected by the temperature, which was earlier than that at the CA test site (Fig. 3B).

### Growth response to climate change

**Figure 4:**
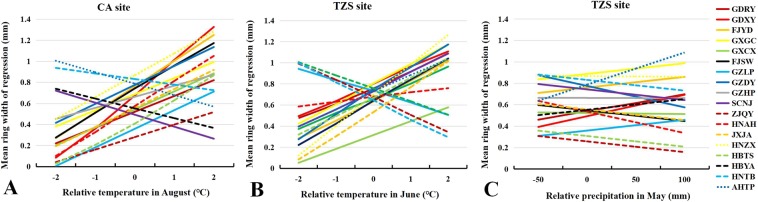
Response of mean RW to major limiting hydrothermal factors: (**A**) Response of mean temperature in August at CA site, (**B**) Response of mean temperature in June at TZS site, and (**C**) Response of total precipitation in May at CA site.

**Figure 5:**
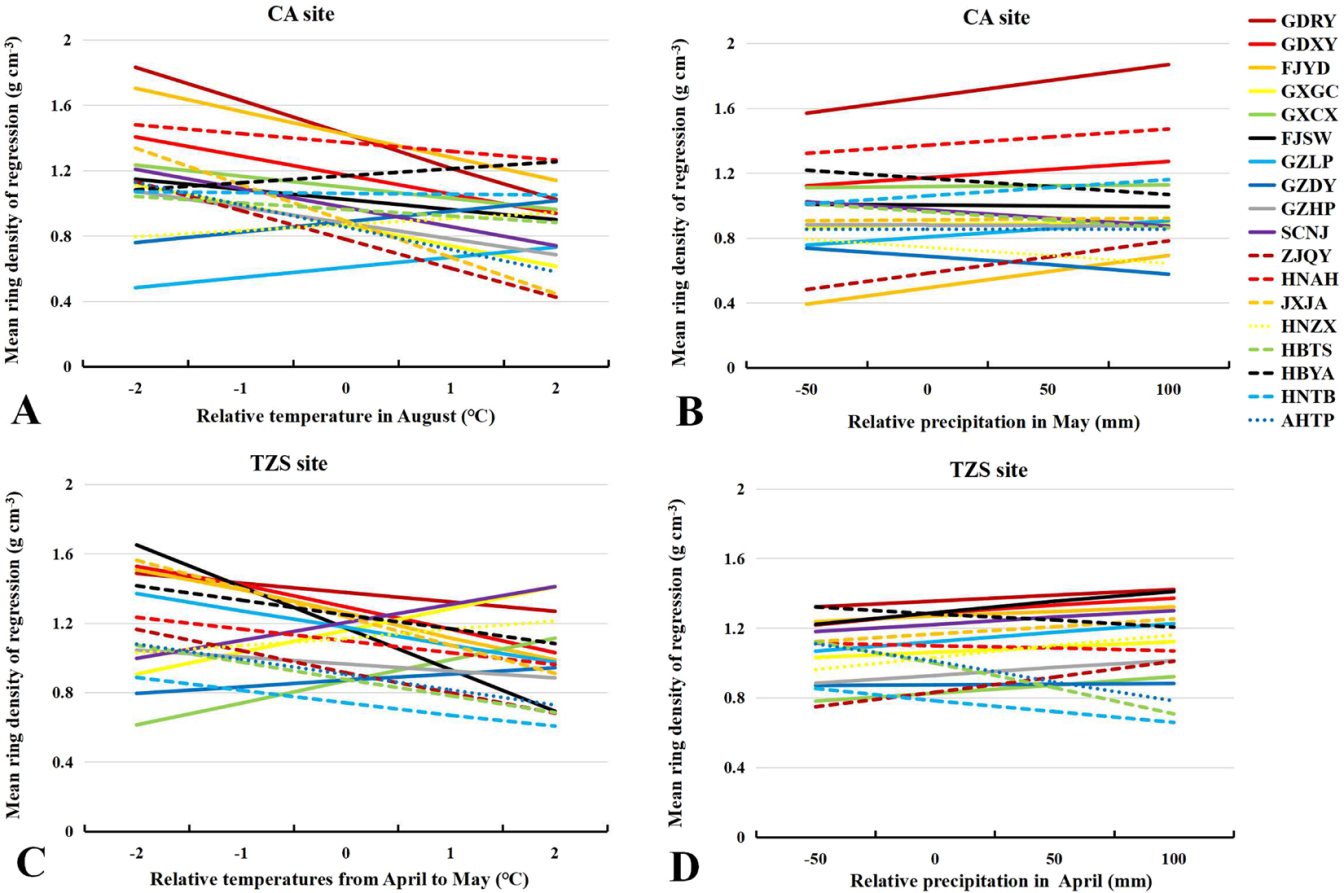
Response of mean RD to major limiting hydrothermal factors: (**A**) Response of mean temperature in August at CA site, (**B**) Response of total precipitation in May at CA site, (**C**) Response of mean temperature in April and May at TZS site, and (**D**) Response of total precipitation in April at TZS site.

The analysis of the linear relationship between climate variables and RW and RD showed that the slope of the linear relationship between various sources was significantly different, which reflected the different responses of various sources to future precipitation and temperature changes (Table S2). At CA test site, the response of RW to the temperature increased in August of a given year varied with the seed source (when all other variables were constant). In general, the RW mostly experienced an increase (GDXY provenance had the largest increase), while RW of SCNJ, HBYA, HNTB and AHTP provenances decreased as the temperature in August increased (Fig. 4A). At TZS test site, RW response to the temperature increase in June of a given year varied with the seed source and mostly increased, although the RW of the GLZP, ZJQY, HBYA and HNTB provenances decreased with temperature increased in June (Fig. 4B). The increasing in precipitation in May of a given year increased the RW of the GDRY, GDXY, FJYD, GXGC, GZLP, HBYA and AHTP provenances (Fig. 4C). At CA test site, RD generally showed a downward trend as the mean temperature in August increased, whereas the RD of the GZLP, GZDY, HNZX and HBYA seed sources increased as the mean temperature in August increased (Fig. 5A). The RD response of the Masson pine provenances was affected by the increase in precipitation in May of a given year (except for the GZDY, SCNJ, HNZX, HBTS and HBYA provenances) (Fig. 5B). At TZS test site, RD generally decreased with the mean temperature increase in April and May (except for the GXGC, GXCX, GZDY, SCNJ and HNZX provenances) (Fig. 5C). The RD response of the Masson pine provenances was affected by the increase in precipitation in April of a given year (except for the HNAH, HNTB, AHTP, HBTS and HBYA provenances) (Fig. 5D).

### Relationships with environmental variables at provenance origin

**Figure 6:**
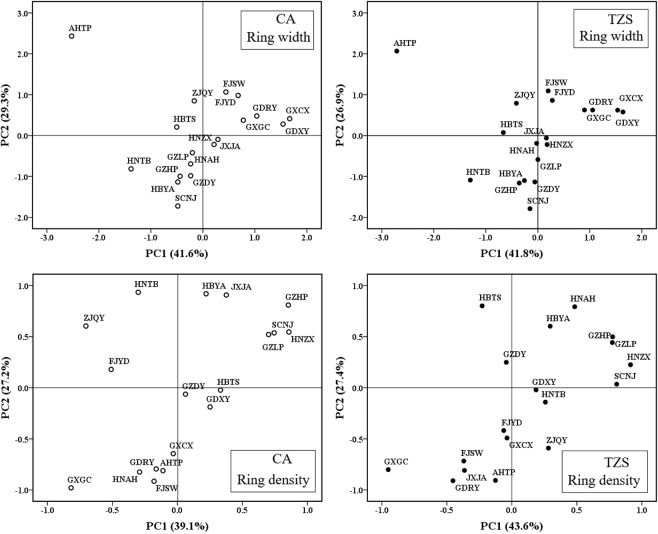
Principal Component Analyses (PCA) plot. PC1 includes latitude and temperature factors, PC2 includes precipitation factors, according to a clustering method involving all provenances (two test sites).

According to the principal components, different provenances were grouped into regions related to climate factors. A scatterplot of the first two principal components was shown in Fig. 6. The first principal component reflected the variable of temperature, and the second principal component reflected the variable of longitude and precipitation corresponding to the load diagram of each factor. In terms of RW, the provenance in the northern and western breeding regions had high dependence on temperature. With the increase of longitude, the provenance in the eastern and southern breeding regions gradually became more dependent on precipitation, while AHTP provenance was an exception here (Fig. 6). In terms of RD, different provenances did not show a common trend in the two planting sites. But, on the whole, the provenances (SCNJ, GZLP, GZHP, HNZX, HBYA and JXJA) in the western and north-central breeding regions performed better in the wood density after introduced to the CA planting sites. After introduced to TZS planting site with abundant precipitation, the provenances (SCNJ, HNZX, GZLP, GZHP, HBYA and HNAH) performed better in the wood density.

The latitude, the mean annual temperature, the mean temperature in January, and the mean annual minimum daily temperature of seed-source origin were significantly related to RW, and they explained between 32%–50% and 31%–36% of the growth variations, respectively. At TZS site, RD showed the significant negatively correlations to average precipitation from May to August. The mean temperature from May to August, the mean annual maximum daily temperature were positively correlated with RD, respectively. At CA test site, the correlation between RD and average temperature from May to August of seed-source origin, was also positive (Table 2).

**Table 2:** Establish regression analysis of RW, RD and main environmental factors of provenance.

Trees round indicators	RW		RD	
	CA	TZS	CA	TZS
Latitude	0.711^*^ (−)	0.600^*^ (−)	0.138 (−)	0.136
Longitude	0.281	0.377 (−)	0.254 (−)	0.193 (−)
Mean annual temperature	0.601^*^	0.561^*^	0.189	0.094 (−)
Mean temperature in January	0.662^*^	0.601^*^	0.176	0.052 (−)
Mean temperature from May to August	0.575^*^	0.436	0.591^*^	0.618^*^
Mean annual maximum daily temperature	0.351	0.387	0.502	0.578^*^
Mean annual minimum daily temperature	0.571^*^	0.594^*^	0.459	0.463
Annual total precipitation	0.509	0.252	0.111	0.197 (−)
Precipitation in January	0.136	0.368 (−)	0.224 (−)	0.058 (−)
Mean precipitation from May to August	0.289 (−)	0.371 (−)	0.309 (−)	0.573^*^ (−)

^*^stands for significant difference level (p < 0.05), *stands for extremely significant level (p < 0.01); (–) stands for a negative relationship.

## Discussion

The study showed that the climatic factors could explain 18.76% and 26.33% of the total variance of the annual RW, which could explain 50.31% and 65.13% of the total variance of the annual RD, and the annual RD was more affected by the climatic environment than the annual RW^3^. The results showed that RW and RD response of provenances varied based on the research of climatic factors. The ranking of provenances on RW and RD changed with cambial age, which might be show the different growth responses of provenances to climatic change. The mean temperature in June and the total precipitation in May was the major climatic factors affecting the Masson pine RW at TZS test site (P < 0.05). TZS test site was located in a relatively dry area in the central and western regions, and the significant impact of precipitation in May on RW might be partly explained by the high temperature in May and June, which also affected RW. Early wood had a high transport capability during its formation^45^. Precipitation from May to August was 550 ml, and relatively dry soil conditions limited the growth of Masson pine at TZS site. In June and July, particularly, which were the most active period of growth for conifers, high summer temperature could greatly reduce soil moisture, thereby affected tree growth. The mean temperature in August was the major climatic factor affected the RW of Masson pine. Precipitation from May to August could reach 810 ml at CA test site; thus, the availability of soil water did not the major factor affecting the growth of RW.

In this study, the major climatic factors affecting RD of Masson pine were the mean temperature in August and the total precipitation in May at CA test site. The main climatic factors affecting RD were the average temperature from April to May and precipitation in April at TZS site. The characteristics of RD showed strong correlations with temperature^46–48^. From April to July, particularly, which were the most active period of growth for conifers, high summer temperature could greatly reduce soil moisture. The needles tended to close stomatal and reduce water loss with transpiration increasing. The affect of precipitation and temperature on radial growth changed gradually with the changing of location and drought degree. For example, at high altitudes, the sensitivity of wood density to temperature would decrease due to climate warming, then turned to the coupling relationship between temperature and precipitation^49,50^. It was important to predict the influence of precipitation on wood density. The total precipitation in April (TZS sites) and May (CA sites) was increased in RD (majority of provenances). Increasing rainfall in early summer, wood formation might have been delayed, which affected the proportion of early-late wood within the tree-ring and increased late wood proportion. The wood density of *P. massoniana* was limited by extreme summer heat in the growing season at CA sites. This was consistent with study findings of significantly positive correlations between wood density and the mean maximum temperature and sunshine hours during late summer and early fall that had been revealed in conifers such as Silver fir (*Abies alba*)^29^. The net photosynthesis of tree affected the duration and rate of diameter growth as well the number and size of the cells formed in the growing season^50^. In the early growing season, the climate is mild, the cambium is active, the radial growth rate is high, and more growth-regulating hormones will be produced in the early growth process. The duration of cell enlargement is relatively long but the duration of cell wall thickening is short, which producing a lot of large, thin-walled cells of earlywood. In the late growing season, given the effective and rapid stomatal control of water loss from transpiration, the formation of photosynthetic products and the storage of carbohydrates are reduced, the speed of cell division falls down, while the speed of enlargement duration of cell increases and thickening duration increases. In the end, some narrow, thick-walled cells of dense latewood are produced^29,50^.

The results also suggested that species from high temperature and low latitude regions had greater RWs compared to species from local and cold sources. One reason: The southern provenances showed faster radial growth, which might be due to adapting to the warm growing environment of seed-source origin. When introduced to the planting site, they still maintained a longer growing period. Another reason: Growth differences between provenances were generally genetically controlled and had certain laws of geographic variatio. For example, the high latitude provenance grows slowly and has strong resistance to snow pressure, wind damage and freezing damage. When low-latitude provenances are shifted to the north, the properties of these provenances, including fast growth and low resistance to snow pressure, freezing damage, and wind damage, are essentially maintained. The growth of trees in southern provenance requires more precipitation compared with that in the northern areas^51–53^. The trend associated with temperature is opposite, while temperature was considered to be an important factor limiting the northward movement of tree species^54^. The study showed that there was a complex relationship between the tree-ring parameters and temperatures during the growing season and across zones. For example, the response of the RW parameter to temperature changed from a positive correlation in high latitudes (late summer) to a negative correlation or no correlation in low latitudes (late spring or early summer)^50^.

However, the geographical genetic variation in wood traits is generally weak or nonexistent. Principal component analysis showed that after introduced to CA planting site, the provenance in the breeding regions in the west and north of China showed better RD in the regions with abundant precipitation, which was gradually higher than that in the breeding regions in the east and south (shown in Fig. 6). The particularity of distribution location and altitude of the breeding regions in the west and north suggested that the growth of these provenances changed the effective use of water in the growing season, while the provenances from the southernmost distribution area were closely related to the change of precipitation in the growing season^29^. We have the following explanation for these results. When many provenances were planted at CA site, the relatively warm and humid climatic conditions of this site benefited the continued formation of late wood because of abundant precipitation and the relatively high temperature during the late growing season. Therefore, late wood density at this site was relatively high. For the southern provenances, because precipitation was inadequate, and temperature was relatively low during the late growing season, late wood density was relatively low due to suppressed growth. After introduced to TZS planting site, the provenances (SCNJ, HNZX, GZLP, GZHP, HBYA and HNAH) showed high dependence on precipitation. The experimental site at TZS had a relatively dry climate, which was similar to what the western and central provenances experienced. Temperature during the late growing season at this site was relatively high, which benefited the continued formation of late wood and resulted in the higher late wood density of provenances in these regions. Because summer precipitation at this site was relatively low, and the temperature during late summer and early fall was also relatively low, the growth of the southern provenances was suppressed, resulting in the lower wood density observed for these provenances.

## Supplementary information

Supplementary Information.
