# Supplementary material for: Joint influence of genetic origin and climate on the growth of Masson pine (Pinus massoniana Lamb.) in China
Source: Sci Rep. 2020 Mar 13;10:4653. doi: 10.1038/s41598-020-61597-9 (PMC7069991; doi:10.1038/s41598-020-61597-9)
Supplement: Supplementary file 1 — Supplementary Information. [file 41598_2020_61597_MOESM1_ESM.pdf]

Joint influence of genetic origin and climate on the growth of Masson pine (*Pinus massoniana* Lamb.) in China

Zhen Zhang[1,2]*, Guoqing Jin[1,2], Zhongping Feng[3], Linshan Sun[4], Zhichun Zhou[1,2]*, Yi Zheng[1,2], & Chengzhi Yuan[1,2]

1. Research Institute of Subtropical Forestry, Chinese Academy of Forestry, Hangzhou 311400, Daqiao Rd 73, Fuyang area, Hangzhou 311400, P. R. China
2. Zhejiang Provincial Key Laboratory of Tree Breeding, Daqiao Rd 73, Fuyang area, Hangzhou 311400, P. R. China
3. Laoshan Forest Farm of Chun'an Country, Zhejiang Province, Chun'an 311700, China
4. Forest farm administration in Hubei taizi mountain, Jingshan, 431822, China
* Correspondence: Zhen Zhang and Zhichun Zhou; Tel.: +86-0571-6331-6172

E-mail:

Zhen Zhang: zhenzh19860516@163.com

Guoqing Jin: 809528714@nefu.edu.cn

Zhongping Feng: 413003320@qq.com

Linshan Sun: djhd@hdu.edu.cn

Zhichun Zhou: zczhou_risf@163.com

Yi Zheng: zhengyi19960922@nefu.edu.cn

Chengzhi Yuan: chengzhiyuan@csuft.edu.cn.

Tab. S 1 Statistical characteristics of residual chronologies calculated with the software ARSTAN

	RW		RD	
	CA	TZS	CA	TZS
Mean sensitivity	0.228	0.225	0.412	0.406
Standard deviation	0.349	0.338	0.187	0.194
Mean inter-serial correlation	0.511	0.475	0.517	0.604
Frist-order autocorrelation	0.003	0.007	-0.014	-0.011
Express population signal	0.879	0.866	0.914	0.902

Tab. S 2 Difference analysis of the linear relationship between RW and RD of provenances and climatic variables

	RW				RD			
	CA		TZS		CA		TZS	
	slope	intercept	slope	intercept	slope	intercept	slope	intercept
Relative rainfall in April (mm)							P<0.001	P<0.001
Relative rainfall in May (mm)			P<0.001	P<0.001	P<0.001	P=0.051		
Average relative temperatures in April and May (°C)							P<0.001	P<0.001
Relative temperature in June (°C)			P<0.001	P=0.274				
Relative temperature in August (°C)	P<0.001	P<0.001			P<0.001	P=0.019		

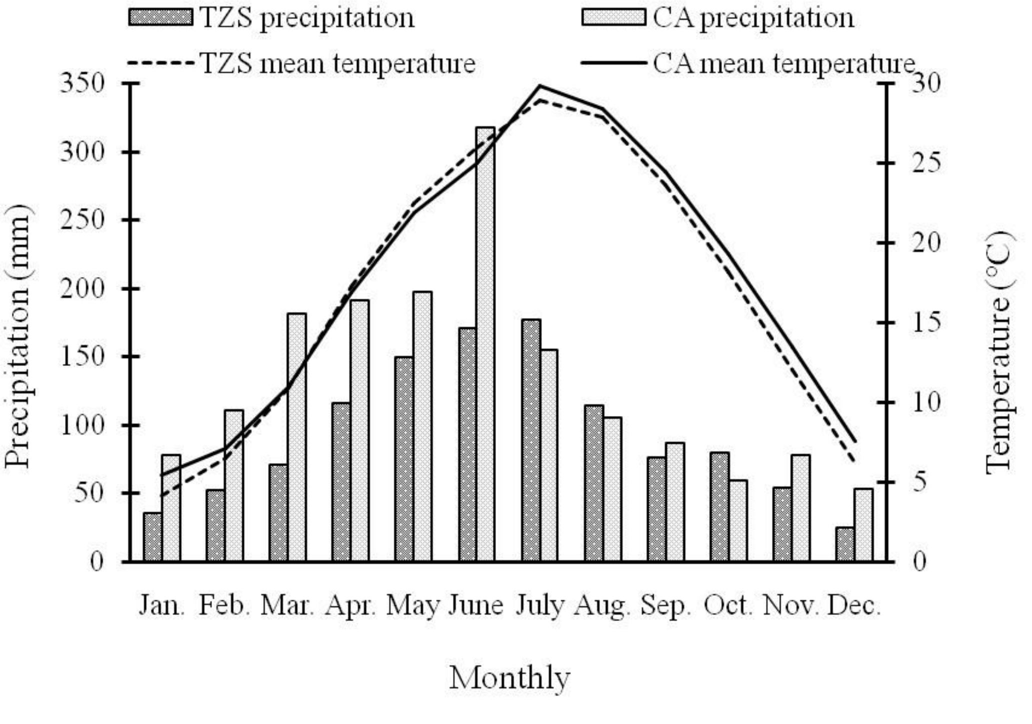

Fig. S1 Monthly mean temperature and total precipitation for the period 1986–2016 at the test sites
